# Evaluating postoperative hernia incidence and risk factors following pelvic exenteration

**DOI:** 10.1007/s00384-024-04638-3

**Published:** 2024-05-08

**Authors:** Nicole Anais Milanko, Michael Eamon Kelly, Greg Turner, Joeseph Kong, Cori Behrenbruch, Helen Mohan, Glen Guerra, Satish Warrier, Jacob McCormick, Alexander Heriot

**Affiliations:** 1 Peter MacCallum Comprehensive Cancer Centre, Department of Surgical Oncology with the University of Melbourne, Melbourne, Australia; 2 Trinity St James Cancer Institute, Dublin, Ireland; 3 Auckland District Health Board, Auckland, New Zealand; 4 Department of General Surgery, Royal Melbourne Hospital, Melbourne, Australia

**Keywords:** Pelvic exenteration, Advanced rectal cancer, Surgical outcomes, Hernia, Postoperative complications

## Abstract

**Abstract:**

Pelvic exenteration (PE) is a technically challenging surgical procedure. More recently, quality of life and survivorship following PEs are being increasingly acknowledged as important patient outcomes. This includes evaluating major long-term complications such as hernias, defined as the protrusion of internal organs through a facial defect (The PelvEx Collaborative in Br J Surg 109:1251–1263, 2022), for which there is currently limited literature. The aim of this paper is to ascertain the incidence and risk factors for postoperative hernia formation among our PE cohort managed at a quaternary centre.

**Method:**

A retrospective cohort study examining hernia formation following PE for locally advanced rectal carcinoma and locally recurrent rectal carcinoma between June 2010 and August 2022 at a quaternary cancer centre was performed. Baseline data evaluating patient characteristics, surgical techniques and outcomes was collated among a PE cohort of 243 patients. Postoperative hernia incidence was evaluated via independent radiological screening and clinical examination.

**Results:**

A total of 79 patients (32.5%) were identified as having developed a hernia. Expectantly, those undergoing flap reconstruction had a lower incidence of postoperative hernias. Of the 79 patients who developed postoperative hernias, 16.5% reported symptoms with the most common symptom reported being pain. Reintervention was required in 18 patients (23%), all of which were operative.

**Conclusion:**

This study found over one-third of PE patients developed a hernia postoperatively. This paper highlights the importance of careful perioperative planning and optimization of patients to minimize morbidity.

**Supplementary Information:**

The online version contains supplementary material available at 10.1007/s00384-024-04638-3.

## Introduction

Pelvic exenteration (PE) is a technically challenging surgical procedure. Over the last two decades, there has been a substantial evolution in the surgical techniques used for exenterative surgery [2, 3]. This is exemplified by the expansion of PE procedures, pushing the boundaries and indications for surgery [2, 4]. Previously thought contraindications including pelvic side-wall infiltration and/or liver metastasis have been challenged [5, 6].

The goal of PE, performed for locally advanced or recurrent pelvic malignancies, is to achieve clear margins, with R0 being an excellent prognosticator of long-term survival [4, 7–11]. However, tumour distortion, previous surgery or neoadjuvant chemo-radiotherapy can make this difficult, resulting in 50 to 60% of procedures achieving R0 resection, especially in the setting of recurrent neoplasms [4, 8, 12]. Depending on the type of cancer, studies have reported 5-year overall survival rates ranging between 28.2 and 66% [5, 13, 14], with postoperative morbidity and mortality between 31.2 and 45.1% [4, 15, 16] and 0.5 and 2% [17] respectively.

More recently, quality of life (QoL) and survivorship following major pelvic surgery are being increasingly acknowledged as important key performance indicator (KPI) outcomes in those undergoing oncological surgery [4, 18]. The focus on QoL has highlighted significant issues in physical, psychological and social implications after PE [18–20]. Major long-term complications range from 2.6 to 32% following multi-visceral pelvic surgery [21] including chronic intra-abdominal abscess/collection, perineal wound issues, stoma-related problems, fistulas and hernias [18, 22]. Literature evaluating postoperative hernia formation in PE cohorts is limited. Furthermore, data regarding flap versus incisional-related issues and their subsequent management are sparse. The aim of this paper is to ascertain the incidence and risk factors for postoperative hernia formation among our PE cohort managed at a quaternary centre.

## Method

A retrospective cohort study examining hernia formation following PE for locally advanced rectal carcinoma (LARC) and locally recurrent rectal carcinoma (LRRC) between June 2010 and August 2022 at a quaternary cancer centre was performed. Specifically, patient characteristics and risk profiles for developing hernias following PE were identified, as well as subsequent management. Data was collected from the Peter MacCallum Cancer Centre (PMCC) beyond the total mesorectal excision database. All patients undergoing PE, who had 12-monthly surveillance imaging (computer tomography, positron emissions tomography-computer tomography and magnetic resonance imaging) and/or clinical evaluation at any time point within the 12-month postoperative period, were assessed for the presence of a hernia (incisional, parastomal, perineal or other) via radiological assessment and independent review. Given the vast geographical scope of the patient cohort, many hernia repairs were considered for repair by external units. However, given the complex surgical history, consultation from PMCC, in particular regarding the risk of disease recurrence and prior surgery, would be sought.

Baseline data was collected including age at diagnosis, gender, body mass index (BMI), site of the primary carcinoma, use of chemo-radiotherapy, smoking status, sarcopenia status, pre-operative biochemical markers (albumin, full blood examination, C-reactive protein, tumour markers) and participation in prehabilitation. In addition, operative details (extent of resection), operative difficulties (where available) and postoperative issues were recorded. Finally, details pertaining to postoperative hernias including type and location of hernia, symptomatology and subsequent hernia management (medical/surgical) were noted. Only data available for ≥ 50% of the cohort were statistically analysed to reduce the impact of information bias.

This study adheres to the STROBE reporting recommendations of the Equator Network.

### Definitions

Total pelvic exenteration (TPE) is defined as the complete *en bloc* resection of the genitourinary viscera, internal reproductive organs (prostate, seminal vesicles, uterus, vagina, cervix), rectum, regional lymph nodes and peritoneum. Anterior pelvic exenteration (APE) includes resection of the bladder with or without internal reproductive organs, and posterior pelvic exenteration (PPE) includes resection of the internal reproductive organs (typically via hysterectomy + / − bilateral salpingooophorectomy with or without vaginectomy) and rectum with preservation of the bladder [11]. A hernia is defined as the protrusion of internal organs through a facial defect [1]. When defining histopathological outcomes, R0 resection is determined by a clear circumferential resection margin (CRM) of more than 1 mm. R1 resection is defined as the presence of microscopic residual disease with a CRM of 1 mm or less, and R2 resection is the presence of macroscopic residual disease [4].

### Endpoints

The primary endpoints were the incidence of hernia formation; factors influencing the incidence of hernia formation including but not limited to BMI, operative approach and resection type and subsequent hernia management. In addition, secondary endpoints assessing hernia complications were also screened for.

### Statistical analysis

Data was analysed using GraphPad Software LLC (Prism 8). Descriptive analysis was undertaken to report variable frequencies. Differences between patient groups were evaluated using the chi-squared (*χ* ^2^) test (for categorical variables), Student’s *t*-test and the Mann–Whitney* U* test as appropriate. Reported intergroup comparisons were significant at the 5% level (*p* < 0.05).

## Results

A total of 243 patients were identified as having undergone a PE procedure over a 12-year period; 150 with LARC and 85 with LRRC were included in the study. Female sex was more common (*n* = 144, 59%) and the median age was 67.5 years (IQR 19). The median BMI for the study cohort was 33 (IQR 8) (Table 1).

**Table 1:** Outline of the patient characteristics from a quaternary centre pelvic exenteration cohort of 243 patients over a 12-year period

**Characteristics (total** ***n*** **= 243)**	
**Age at diagnosis** (years)	
Median (IQR)	60 (18.99)
**Gender**: *N* (%)	
Female	144 (59.23)
Male	99 (40.74)
Sex ratio (M to F)	0.69
**BMI** (kg/m^2^) (IQR)	33 (8)
**Neoadjuvant therapy**: *N* (%)	
Yes	230 (94.65)
No	12 (4.94)
Unknown	1 (0.41)

A total of 79 patients (32.5%) were identified as developing a hernia, 52 stoma-related hernias, 17 incisional hernias, seven multi-site hernias, two with hernias at vertical rectus abdominis myocutaneous (VRAM) flap site and one perineal hernia (Table 2). Male sex was more common (*n* = 50, 63%) and the median age was 67 years (IQR 15). The median BMI of those who developed hernias was 33 (IQR 8). Pre-operative albumin, full blood examination (FBE), C-reactive protein (CRP) and carcinoembryonic antigen (CEA) were available for 73%, 79%, 61% and 34% of the cohort respectively (Table 3).

**Table 2:** Baseline data in patients with hernia formation

**Characteristics (*n*** **= 79)**	
**Age** (years)	
Median (IQR)	67 (15)
**BMI** (kg/m^2^) (IQR)	28 (8.75)
**Gender**: *N* (%)	
Female	29 (36.71)
Male	50 (63.29)
**Neoadjuvant therapy**: *N* (%)	
Chemo-radiotherapy	2 (2.53)
Radiotherapy alone	5 (6.33)
Chemotherapy alone	74 (93.67)
**Smoking status**: *N* (%)	5 (6.33)
Yes	11 (13.92)
Ex	33 (41.77)
No	26 (32.91)
Unknown	9 (11.39)
**Sarcopenia status**: *N* (%)	
None	
Mild	
Moderate	
Severe	
**Pre-operative biochemical markers: median** (IQR)	
Albumin	35 (6.75)
Full blood examination	126 (26.5)
C-reactive protein	8.15 (21.05)
Carcinoembryonic antigen	2.6 (6.3)
**Prehabilitation**: *N* (%)	
Yes	47 (59.50)
No	28 (35.44)
Unknown	4 (5.06)
**Site of primary carcinoma**	
Pelvic sarcoma	1 (1.27)
Caecal	1 (1.27)
Sigmoid	2 (2.53)
Rectal	63 (79.75)
Anal	7 (8.86)
Prostate	2 (2.53)
Bladder	1 (1.27)
Uterus	1 (1.27)
Vagina	1 (1.27)

**Table 3:** Effect of pre-operative and intraoperative features on hernia formation in 243 patients

	***Hernia formation***		***P-value***	***Confidence interval***
	Yes (*n* = 79)	No (*n* = 164)		
**Age** (years), median (IQR)	66 (15)	62 (21)	*p* = 0.06	− 0.2260 to 7.112
**BMI** (kg/m^2^) (IQR)	28 (8.75)	25 (7)	*p = *0.02*	1.012 to 4.467
**Surgical approach**: *N* (%)				
Open	73 (92.41)	153 (93.29)	*p* = 0.55	
**Type of pelvic exenteration surgery**: *N* (%)			*p* = 0.55	
Total	38 (48.10)	62 (37.80)		
Modified	41 (51.90)	87 (52.05)		
**Bone resection**: *N* (%)			*p* = 0.89	
Yes	36 (45.57)	73 (44.51)		
No	43 (54.43)	91 (55.49)		
**Side-wall resection**: *N* (%)			*p = *0.05*	
Yes	24 (30.38)	72 (43.90)		
No	55 (69.62)	92 (56.10)		
**Flap reconstruction**: *N* (%)			*p = *0.04*	
Yes	45 (56.96)	70 (42.68)		
No	34 (43.04)	94 (57.32)		
**Flap type**: *N* (%)				
ALT	5 (11.11)	10 (6.10)		
IGAP	3 (6.67)	1 (0.61)		
IGAM	31 (68.89)	55 (33.54)		
VRAM	6 (13.33)	3 (1.83)		

*ALT* anterolateral thigh, *IGAP* inferior gluteal artery perforator, *IGAM* inferior gluteal artery myocutaneous, *VRAM* vertical rectus abdominis myocutaneous

*Statistically significant difference (*p*<0.05)

Patients who developed postoperative hernias were on average older than those who did not (67 years vs. 62 years, *p* = 0.06). Proportionally, a greater percentage of male patients developed postoperative hernias (63% vs 57%) compared to female patients (37% vs. 43%), though these differences were not statistically significant. Those who developed hernias had a higher BMI (*p* = 0.02), with a median value of 28 (IQR 8.75) compared to those who did not with a median BMI of 25 (IQR 7).

Expectantly, those undergoing flap reconstruction had a lower incidence of postoperative hernias. Of the total cohort, 115 individuals underwent flap reconstruction. Of these, 45 individuals (39%) developed hernias and 70 individuals (61%) did not (*p* = 0.04). Types of flaps performed included anterolateral thigh (ALT), inferior gluteal artery perforator (IGAP), inferior gluteal artery myocutaneous (IGAM) and vertical rectus abdominis myocutaneous (VRAM) flaps.

There was not a statistically significant difference regarding the incidence of postoperative hernias when analysing the surgical approach (Table 4). Total pelvic exenteration was the most common approach, followed by PPE. There was a statistically significant difference noted in hernia rates when assessing the type of tissue resected, specifically side-wall resection. Of the 96 patients who underwent side-wall resection, 72 (75%) did not develop postoperative hernias (*p* = 0.05).

**Table 4:** Qualifying postoperative and hernia data in the PE cohort of 243

**Number of patients (*n*** **= 243)**	
**Median duration of hospital stay** (days)	20 (IQR 20)
**Mortality within 30 days**: *N* (%)	
Yes	0 (0.00)
No	226 (93.00)
Unknown	17 (7.00)
**Evidence of hernia at 1-year follow-up**: *N* (%)	
Yes	36 (14.81)
No	197 (81.07)
Unknown	1 (0.41)
Not applicable	9 (3.70)

**Number of patients (*n* = 79)**	
**Type of hernia**: *N* (%)	
Incisional	16 (20.25)
Perineal	1 (1.27)
Parastomal	56 (70.89)
Flap	2 (2.53)
Multi-site	4 (5.06)
**Location of hernia**: *N* (%)	
Colostomy	45 (56.96)
Ileostomy	4 (5.06)
Ileal conduit	3 (3.80)
Incisional	17 (21.52)
Perineal	1 (1.27)
Flap (VRAM)	2 (2.53)
Multi-site	7 (8.86)
**Operative hernia repair**: *N* (%)	
Yes	18 (22.78)
No	54 (68.35)
Pending	3 (3.80)
Unknown	4 (5.06)
**Symptomatic hernia**: *N* (%)	
Yes	6 (7.59)
No	73 (92.41)
**Hernia complications**: *N* (%)	
Yes	1 (1.27)
No	78 (98.73)

Those patients deemed *not applicable* for CT evidence of hernia at 1-year follow-up were deceased prior to evaluation

*VRAM* vertical rectus abdominis myocutaneous

Seventeen patients from the entire PE cohort were deceased prior to the 1-year follow-up at which patients were evaluated for postoperative hernias via CT. At the 1-year follow-up, 14% of patients had CT evidence of postoperative hernias.

Of the 79 patients who developed postoperative hernias, 16.5% reported symptoms. The most common symptom reported was pain (9%), followed by difficulty fitting the stoma appliance/dressing (5%), and one patient reported ulceration and leakage around the parastomal hernia site. Reintervention was required in 18 patients (23%), all of which were operative in nature, with three patients (4%) currently awaiting surgical hernia repair. Only one of 79 postoperative hernia patients experienced a complication directly relating to their hernia. Parastomal hernias made up the majority (71%) of hernia presentations, with para-colostomy hernias being the most common site of occurrence at 57%. Three patients experienced postoperative complications following hernia repair including iatrogenic bowel injury requiring repair with ileocolic anastomosis, as well as hernia recurrence and chronic mesh infection.

## Discussion

Pelvic exenterations remain an evolving, radical procedure. The last decade has seen improvements in radiology, patient selection and pre-operative optimization including prehabilitation [23]. It continues to have a considerable impact on patient QoL, both physically and psychosocially.

This study found over one-third of PE patients developed a hernia postoperatively, with 16.5% of these patients reporting chronic symptoms. Interestingly, flap-related hernias, all of which were VRAM flaps, occurred in 1% of the total cohort (*n* = 243) and made up 3% of the postoperative hernia cohort (*n* = 79). Of these, all but one, which was a perineal hernia, were at the donor site. This may be due to the lack of additional mesh used when closing VRAM flaps, a technique superseded by IGAM flaps, which have been shown to have more favourable patient outcomes [24]. One-fifth of those who developed postoperative hernias proceeded to have further elective surgical management with a success rate of 90%. Of note, only one patient experienced a severe complication requiring emergency reduction of their parastomal hernia after presenting with a large hernia around their colostomy site containing omentum with haemorrhagic ischaemia of the small bowel which, after reduction, was viable with rewarming. 

This study highlights the difference in clinicopathological variables, including an elevated BMI and not undergoing flap reconstruction, in the formation of postoperative hernias in the PE cohort. It was unsurprising that an elevated BMI was greater in those with postoperative hernias given the established relationship between increased abdominal pressure due to obesity and hernia formation [25]. Interestingly, side-wall resection appeared to negatively correlate with the incidence of postoperative hernias. There was an association between side-wall resection and flap reconstruction.

Using data previously compiled by the PelvEx Collaborative, the cost of performing a PE is approximated to range from US$13,230 to US$43,950, though this does not include the cost of reintervention for postoperative complications [26]. As well as the financial implications, this paper highlights the importance of careful perioperative planning and optimization, including prehabilitation which 58% of patients underwent, and a multidisciplinary surgical sub-specialty approach with urological, plastic and gynaecological surgeons as needed. The morbidity of hernia formation can exacerbate an already steep learning curve. The most common symptoms experienced were pain, ranging from discomfort to severe, followed by difficulty fitting the stoma appliance and stoma leakage with ulceration. Sufficient access to support is critical in addressing this, which includes ward-based education developing patient, family and carer independence, symptom safety netting, acute and long-term surgical follow-up, community medical and stomal support and financial assistance where eligible.

This study is limited by the retrospective design. This includes the heterogeneity of the study population including pre-morbid function and status, different neoadjuvant treatment regimens and operative approach. Furthermore, the 12 years over which patients were evaluated have seen significant changes in the surgical techniques applied to PEs such as increased radicality and the transition from a palliative to curative purpose with improved long-term survival rates, particularly among patients with LARC [23]. Other limitations include inconsistencies in the data gathered across the cohort, with smoking status, biochemical markers, sarcopenia, information on prehabilitation participation and details of neoadjuvant therapy evaluated for the postoperative hernia population only. Whilst detailed information on surgical technique such as approach, location and type of resection and flap reconstruction was available, further details unavailable to the authors regarding size and location of incision site and closure would have also been valuable modifiable parameters to evaluate when guiding best practice specifically regarding the prevention of postoperative hernia formation. It is also important to acknowledge that the highly specialized nature of PEs confines the external validity of this paper to those centres offering PEs. Fortunately, however, in appropriate patient cohorts, this is applicable globally.

Exenterative surgery has profound implications on QoL with patients, families and carers all important stakeholders to consider. Counselling patients on postoperative morbidity is vital to facilitate best patient care, particularly given some outcomes and complications have long-term effects. This paper highlights the real-world incidence of developing a hernia after a PE, and underscores the importance of considering risk factors and mitigation strategies during perioperative planning.

## Supplementary Information

Below is the link to the electronic supplementary material.

Supplementary file1 (PDF 92 KB)

## Data Availability

All data supporting the findings of this study are available within the paper. The data that support the findings of this study are not openly available due to reasons of sensitivity and patient confidentiality.
